# Varicella Zoster Virus Meningitis with Absence of Rash in an Immunocompetent Child

**DOI:** 10.1155/2021/2081270

**Published:** 2021-09-30

**Authors:** Mariam Lagrine, Karima El fakiri, Noureddine Rada, Ghizlane Draiss, Nabila Soraa, Mohammed Bouskraoui

**Affiliations:** ^1^Pediatric a Department, University Hospital Med VI, Marrakesh, Morocco; ^2^Biology Department, University Hospital Med VI, Marrakesh, Morocco

## Abstract

Only a few cases in the literature have ever reported the reactivation of the varicella zoster virus (VZV) in children especially in the case of immunocompetent patients. It is an uncommon situation that may lead to several neurological complications. We report varicella zoster virus (VZV) meningitis in a 14-year-old healthy boy with no antecedent of rash. On his cerebrospinal fluid (CSF) examination, VZV DNA was detected. The rapid HIV test was negative. The treatment using acyclovir (20 mg/kg/8h) was effective, and the child's clinical condition rapidly improved.

## 1. Introduction

Chickenpox is an infection caused by the varicella zoster virus. It causes severe complications in adults and adolescents. Immunodeficient people are particularly at a higher risk of developing primary multisystemic infection with pulmonary, neurological, and/or hepatic complications. Reinfection is probably more common than previously thought, but its management is similar to primary infection. Reactivation, commonly known as shingles, may also manifest in the form of meningitis, vasculopathy, and disseminated shingles in immunodeficient patients.

We report a case of a healthy child with viral meningitis due to VZV reactivation. The rare isolation of VZV in CSF in immunocompetent children and the absence of skin rash make this case interesting.

## 2. Case Report

A 14-year-old healthy boy presented with a headache, vomiting, and a fever for over 5 days. He also had neck stiffness. He had no past history of skin rash and was neither recently vaccinated nor in contact with infected individuals. He had chickenpox at 18 months and had completed all his vaccines according to the Moroccan national vaccination program.

On physical examination, the patient was febrile at 38.1°C; he had nuchal rigidity, without any abnormal neurological signs or skin rash. The vital signs were as follows: pulse rate was 64 beats/minute, respiratory rate was 20 beats/minute, and SpO_2_ was 99% on room air. Laboratory tests revealed normal white blood cells and platelet count (WBC: 9830/mm and PLT: 277.000/mm) and normal C-reactive protein (0.3 mg/L).

Due to the presence of the meningeal syndrome, cerebrospinal fluid (CSF) was drawn and analyzed. It contained 60/mm^3^ of WBCs and 80% mononuclear; CSF protein was 27 mg/dl, and CSF glucose was 77 mg/dl (serum glucose: 115 mg/dl). He was treated with intravenous ceftriaxone awaiting bacterial culture.

On the second day of evolution, the child still had the headache. Results of bacterial culture came out negative, and the polymerase chain reaction (PCR) in the CSF for bacterial and viral identification revealed varicella zoster virus (VZV). The intravenous ceftriaxone was stopped, and acyclovir was started at a dose of 20 mg/kg every 8 hours for a duration of 10 days.

On the 3rd day of treatment with acyclovir, the patient became apyretic and asymptomatic. A rapid HIV test was requested in view of the PCR results; it also came out negative.

## 3. Discussion

The varicella zoster virus (VZV) belongs to the family of Herpesviridae, a double-stranded DNA virus enclosed in a cubic symmetry nucleocapsid surrounded by a seed coat and an envelope. Being a fragile virus, it is quickly degraded by heat and loses its infectious power after a freeze-thaw cycle. It is fundamentally dermotropic; the virus has neurotropism like the herpes simplex virus (HSV), and it can also remain latent in sensory ganglia. The reactivation of the virus leads to clinical manifestations of shingles and sometimes to a disseminated disease in immunocompromised children.

Children who are in contact with varicella in the first year of life may have zoster during childhood, but there are no publications to support this and further demonstrate that these children are at a higher risk for VZV associated with central nervous system (CNS) manifestations [1].

The CNS manifestations, especially in meningitis caused by VZV, might be as a result of both primary and reactivated disease or might be the cause of vaccine strain reactivation in immunocompetent children [2].

Meningitis in varicella zoster is rare. A report by Galil et al. [3] showed that, in a series of 859 adults, only 0.5% presented with herpes zoster infection. Pahud et al. [4] reported meningitis at 50% in a series of 26 patients presenting with VZV PCR-positive in cerebrospinal fluid in patients aged between 12 and 85 years (median, 46 years).

Other cases report that meningitis in children is usually associated with skin rash due to the past history of varicella primary infection or due to the reactivation of the VZV [1, 5–7]. Sometimes, the CNS manifestation may appear before the skin rash in varicella and in herpes zoster [2, 8].

Aseptic meningitis due to VZV is usually associated with varicella in immunocompromised children. The absence of rash is uncommon in immunocompetent children according to a report from a hospital in southern Iran in which 2 cases out of 30 were positive for PCR of CSF: VZV was isolated from these samples which were collected from children aged between 2 months and 15 years presenting with aseptic meningitis [9]. In adults, only one case was reported in a woman presenting with aseptic meningitis with hypoglycorrhachia without skin rash [8].

The uniqueness of our case lies in the fact that this incident of aseptic meningitis which affected an immunocompetent child was due to VZV reactivation, but the child did not present any skin lesions. In pediatrics, it is rare that the PCR in cerebrospinal fluid can isolate VZV in case of unexplained aseptic meningitis.

We conclude that aseptic meningitis is one of the rare complications of VZV reactivation without skin rash and PCR is the first go to diagnostic test to identify the etiology of the viral meningitis. In case of meningitis due to VZV, eventual treatment with acyclovir intravenously for 7 to 10 days has been recommended for immunocompetent children [1].

## Data Availability

The data used to support the findings of this study are available from the corresponding author upon request.
